# How Can On-Road Hazard Perception and Anticipation Be Improved? Evidence From the Body

**DOI:** 10.3389/fpsyg.2019.00167

**Published:** 2019-02-01

**Authors:** Mariaelena Tagliabue, Michela Sarlo, Evelyn Gianfranchi

**Affiliations:** Department of General Psychology, University of Padua, Padua, Italy

**Keywords:** hazard perception, moped-riding simulator, learning generalization, implicit learning, skin conductance response

## Abstract

The present research is aimed at investigating processes associated with learning how to drive safely. We were particularly interested in implicit mechanisms related to the automatic processing system involved in decision making in risky situations (Slovic et al., 2007). The operation of this system is directly linked to experiential and emotional reactions and can be monitored by measuring psychophysiological variables, such as skin conductance responses (SCRs). We focused specifically on the generalization of previously acquired skills to new and never before encountered road scenarios. To that end, we compared the SCRs of two groups of participants engaged, respectively, in two distinctive modes of moped-riding training. The active group proceeded actively, via moped, through several simulated courses, whereas the passive group watched video of the courses performed by the former group and identified hazards. Results indicate that the active group not only demonstrated improved performance in the second session, which involved the same simulated courses, but also showed generalization to new scenes in the third session. Moreover, SCRs to risky scenes, although present in both groups, were detectable in a higher proportion in the active group, paralleling the degree of risk confronted as the training progressed. Finally, the anticipatory ability demonstrated previously (and replicated in the present study), which was evident in the repeated performance of a given scenario, did not seem to generalize to the new scenarios confronted in the last session.

## Introduction

In the field of road safety research, several studies have gathered indirect and/or direct evidence that supports the idea of the crucial role played by hazard perception in predicting crash likelihood (Horswill, 2016a). As Horswill (2016b) noted, conducting hazard perception research is not feasible in actual on-road situations, as exposing humans to hazards and the consequent potential dangers for the purpose of research raises ethical and other related issues. For this reason, considerable efforts have been devoted to creating adequate tools for measuring this skill in safe contexts.

Two principal methods are currently employed to improve hazard perception among learners (i.e., unlicensed drivers) and novice drivers: one involves watching video clips during which the learners must identify onscreen hazards and the other relies on engaging learners in virtual driving experiences via simulators that administer a variety of hazardous scenarios. The first method is a component of traditional licensing programs in such countries as England and Australia, and the second method has been a compulsory part of Japan’s licensing program for years (Haworth et al., 2005).

Recently, studies aimed at investigating the efficacy of these training methods have demonstrated a positive correlation between a learner’s increased engagement in the task, the efficacy of the training, and the likelihood that the improved ability will translate to actual on-road performance; the degree of engagement is itself increased through the requirements conveyed by instructions and the quality of the feedback delivered (Horswill, 2016b; Horswill et al., 2017). Torres et al. (2017) have provided a coherent account of their finding that contingent negative feedback (i.e., losing license points after unsafe decisions in risky scenarios), delivered in response to decisions about whether or not to brake after the presentation of static on-road scenes, yielded to faster and safer decisions. Moreover, Megías et al. (2017) showed that performance in a moped-riding simulator became safer (in terms of the number of accidents, average speed, average time exceeding the speed limit) among participants trained via a feedback learning task that delivered emotional feedback (i.e., pictures of real accidents) with negative valence. Specifically, the learning task consisted of deciding whether it was appropriate to brake, given a set of traffic images. Participants who received negative emotional feedback in 50% of the trials in which they decided not to brake upon being presented with a risky scenario (i.e., risky decision-making behavior) demonstrated safer behavior in the subsequent moped-riding simulator test.

In this regard, one important consideration is that when learning involves simulation driving, feedback is embedded within the task; to wit, the driver directly experiences the consequences of risky decisions in the form of the dangers incurred (i.e., crashes or near misses). The use of simulators intrinsically involves a degree of uncertainty with regard to the extent that such results could be generalized to an on-road context (de Winter et al., 2012; Rosenbloom and Eldror, 2014), given that definitive proof would require prolonged monitoring of very large samples and the fact that the likelihood of crash occurrence is relatively low throughout the general population (although regarded as too high to satisfy the safety standard requirements). Nonetheless, some indirect evidence already collected indicates that driving styles recorded via simulator resemble, to some extent, the corresponding on-road driving styles (Goode et al., 2013; Meuleners and Fraser, 2015; Branzi et al., 2017). In particular, the behavioral changes, observed during simulated driving tasks as training progresses, toward safer driving behaviors (i.e., reduction of the probability to incur a crash) are comparable to on-road behaviors, and learning acquired via simulated driving persists over time (Vidotto et al., 2011, 2015).

Moreover, the advantage of using driving simulators to deliver feedback resembling real-life consequences is intrinsically linked to the interactive nature of the virtual environment in the sense that the world changes in response to the driver’s behavior (de Winter et al., 2012). Thus, we can assume that quality, degree of contingency, and valence of feedback are crucial factors contributing to the improvement of hazard perception. Consequently, simulated driving should be regarded as more effective at promoting a defensive driving style than passive forms of training because the feedback provided to the trainee is coherent and directly linked to his or her behavior. Further, it can be hypothesized that simulators are potentially more engaging from an emotional perspective, as the first-hand experience of a virtual accident might, in principle, be recognized as more emotionally intense than the simple imposition of a virtual penalty. Moreover, experiencing a car crash for which the driver is personally responsible might produce a more vivid experience than watching images of a crash that is caused by and affects other people.

The aforementioned dynamic characteristic of simulations is thereby related to another advantage yielded by this kind of technology. We refer to the extent to which simulations involve the potential exposure of the driver to hazards (de Winter et al., 2012). First, the simulated nature of this exposure limits the ethical issues that would otherwise plague such research. Consequently, it enables the reproduction of road conditions characterized by the highest statistical probability of accidents occurring, and in which the hazardous element develops “spontaneously” and directly via the specific way in which each driver behaves in each condition. Thus, the use of driving/riding simulators ensures both the standardization of experimental (or training) conditions and the modulation of the hazard degree, pursuant to the specific driving style of each learner.

Another feature that has emerged as a critically important variable is the emotional involvement associated with participation in hazard perception ability training. Currently, a consensus has been reached about the claim that human beings respond to risks in ways that are often not supported by rational rules. According to Slovic et al. (2007), decisions in risky situations are made via an automatic processing system that relies on emotion and experience, which is mostly irrational and more rapid than the controlled processing system.

The mechanism to which Slovic et al. (2007) explicitly referred is the one accounted for by Bechara et al. (1994) and Damasio (1994). By comparing the performances of healthy individuals and ventromedial prefrontal patients in a decision-making task [i.e., the Iowa Gambling Task (IGT)], Bechara et al. (1994) observed that healthy participants develop, over the course of the task, an anticipatory ability that prevents them from choosing from decks that, in previous trials, had yielded losses that outweighed their gains. This occurs via psychophysiological activation [measured through skin conductance responses (SCRs)] that function as signals to alert participants that they are approaching a deck of cards that they have previously experienced as disadvantageous. This ability cannot develop in patients with damage to the ventromedial prefrontal cortex who, consequently, show smaller gains (if not explicitly greater losses) by the conclusion of the task. Interestingly, decision-making ability, as measured by the IGT, seems to interact with other personality variables that have demonstrably correlated with dangerous driving styles (Gianfranchi et al., 2017a); to wit, it has been shown that IGT performance and sensation-seeking behavior interact in contributing to simulated motorcycle riding style (Gianfranchi et al., 2017b).

Within this framework, a reasonable amount of evidence has been collected on the psychophysiological correlates of hazard perception and reactions in the context of driving. For instance, in one of the first studies, Helander (1978) showed that, in situations that required the use of brakes, participants demonstrated electrodermal responses that were interpreted as deriving from mental activity preceding muscular contraction and, consequently, brake activation.

In a more recent study, Kinnear et al. (2013) demonstrated that, when participants were shown video clips of hazardous road scenarios and asked to identify oncoming dangers, learners and novice drivers exhibited a smaller proportion of anticipatory SCRs than experienced drivers. Moreover, focusing on novice drivers, Tagliabue and Sarlo (2015) showed that employing a driving simulator ensures greater emotional involvement in the task, relative to traditional methods that consist exclusively of exposure to video clips (passive training), as demonstrated by a larger proportion of SCRs. Further, the administration of the same road-simulated scenarios twice, the first a week before the second, has been proven to lead to earlier SCRs (Tagliabue et al., 2017), suggesting improved anticipatory ability, in line with the somatic marker hypothesis (Damasio, 1994): When people encounter the same situation that previously led to an emotional reaction, they experience a reactivation of the same emotions, via their “bodily reactions,” which provide them with an advance signal that alerts them to the oncoming risk.

Given the importance and potential implications of such results for driver training programs, it is worth considering two crucial issues arising from the aforementioned evidence. The first concerns the effectiveness of passive training in improving the anticipatory recognition of hazards. The second deals with the need to understand whether or not this anticipation generalizes to new and different scenarios. Both issues have important implications for the design of future programs for learners and novice drivers.

## The Study

The present work is part of a project aimed at investigating the processes involved in hazard perception improvement induced by the Honda Riding Trainer (HRT), a moped simulator. The HRT has proven to be a useful tool for promoting a defensive riding style among novice teenaged riders (Vidotto et al., 2011) by training the attentional skills (Tagliabue et al., 2013) that presumably underlie successful hazard detection; further, the demonstrated improvement in riding style persists for over a year (Vidotto et al., 2015). The HRT has been used to investigate a variety of the processes that underpin driving abilities, such as mental workload during driving, cognitive resources needed to respond to in-vehicle warning systems, and patterns of gaze exploration (Di Stasi et al., 2009, 2010, 2011). Starting from these results, we wanted to investigate the processes underlying hazard perception that could feasibly account for the improvements observed.

As noted above, earlier studies indicated that participants who undertook active training in the form of simulated moped-riding courses demonstrated a greater SCR percentage than participants who engaged in passive training by identifying hazards on these same courses upon request. Further, by comparing the SCRs of active participants who tackled the same courses in the HRT in two sessions (1 week apart) it was shown that SCR onset decreased during the second session, indicating that the first experience negotiating the hazardous scenarios enabled the riders to recognize these now-familiar hazards some 3 m of (virtual) road before (Tagliabue and Sarlo, 2015; Tagliabue et al., 2017).

Three central questions remain open, and these revolve around whether: (a) the anticipatory response does, in fact, generalize to new hazardous conditions; (b) the improvement in anticipatory ability related to hazard detection and reaction differs on the basis of whether the training is active or is passive; and (c) the overall profile of the performance, besides the probability that a crash will occur, is impacted by the active training involving the simulator or the passive training consisting of viewing video clips of hazardous scenarios.

Concerning the third point, in the above-mentioned studies that employed the HRT, improvement in performance was essentially measured by calculating the percentage of accidents. However, another crucial aspect of road safety involves learning to drive in a way that facilitates averting hazard development. To wit, learners may demonstrate a lower percentage of crashes either because they have learned to execute certain last-minute maneuvers designed to avoid impending collisions or because they have learned to drive in a generally safer way. This issue can be addressed by analyzing the level of potential hazards that develop during active training. Indeed, the HRT simulator facilitates such an analysis by providing a score for each potentially hazardous scene based on the degree of hazard development: when the rider behaves in a way that is totally safe, the scene receives a score of A; when the rider relies on mildly unsafe maneuvers (e.g., moderate violations of the speed limit, slightly sudden braking, stopping with insufficient headway), the scene receives a B score; when the rider executes seriously unsafe maneuvers (e.g., strong sudden braking that results in a concrete risk of losing control of the vehicle, stopping dangerously close to the preceding vehicle), the scene is assigned a score of C, generally reflecting conditions that resemble near-misses; finally, a score D corresponds to scenes in which an accident actually occurs.

By analyzing how the scores collected during training reflect a trend of behavioral change toward generally safer behavior, it may be possible to acquire further information on the mechanisms underlying the learning process.

To that end, in the present work, participants in the active group of the study conducted by Tagliabue et al. (2017) were assigned to complete a third training session consisting of the administration of six new courses to investigate issues related to the generalization of learning (aim a). A new group of matched participants (passive group) was recruited, and they engaged in training by watching the scenes attempted by the active group, and identifying hazards (aim b).

Both behavioral and SCR data were collected to measure how participants learned to detect and anticipate hazards. We formulated three hypotheses, as follows:

(1) If the behavioral effects of learning led to an overall safer riding behavior and this generalized to new scenes, we expected to observe, in the third session, not only a lower percentage of accidents, but also a reduction in C scores and an increase in A and B scores.
(2) If the improvement of implicit hazard perception was triggered by active driving more than it was by passive video clip viewing, the latter group would demonstrate a distinct pattern of psychophysiological activation (as measured by the SCR percentage), compared to the active group.
(3) If the anticipatory ability acquired by the participants that actively ride the moped simulator really generalized to road scenes not previously encountered, we expected SCR onset to occur earlier in the third session than in the first session. If, as the somatic marker hypothesis predicts, previous exposure is required for the development of anticipatory responses, then in the third session, the SCR onset would be similar to that recorded in the first session.

### Methods

#### Participants

Thirty-eight undergraduates at the University of Padua were included in the present study. Data from one male participant of the control group were excluded from analyses due to electrode malfunction during skin conductance recording in the third session. Consequently, the data from the matched participant of the experimental group were also eliminated. Thus, the final sample included 36 undergraduates (18 females and 18 males; mean age 19.47 years; range 18–24 years). The experimental group was the same as used in the study conducted by Tagliabue et al. (2017), with the inclusion of three new participants to attain the sample size of 18 participants. The other 18 participants, assigned to the control (passive) group, were all new to this set of studies. All participants were novice drivers/riders; they held a driver’s license for no longer than 3 years (range 5–36 months; mean 9.8 months). Nine students had a riding license, but reported road exposures under 5,000 km. The experimental and control groups were balanced for age (mean age = 19.88 and 19.05 years, respectively) and gender (nine males and nine females in each group).

The procedure included three experimental sessions. The task assigned to the experimental group was to ride a motorcycle simulator along some virtual courses. Participants in the control (passive) group were asked to detect hazards while they watched videos of the experimental (active) participants riding the simulators through the virtual courses.

All participants had normal or corrected-to-normal vision. They were paid €39 for their participation. The study was approved by the Ethical Committee for the Psychological Research of the University of Padua.

#### Apparatus and Stimuli

**FIGURE 1:**
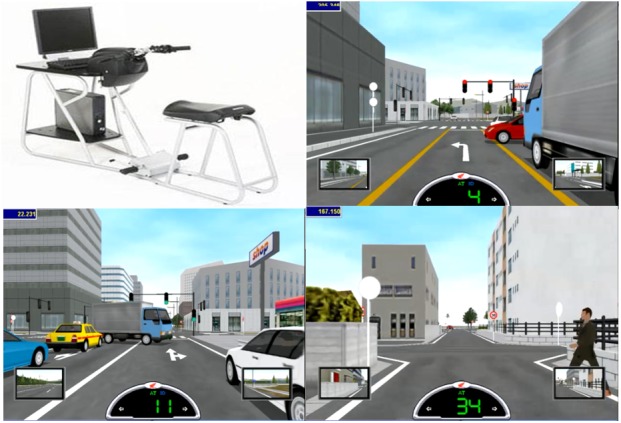
The HRT simulator used in the present study (top-left panel) and three examples of risky scenes.

The HRT is a two-wheeled-vehicle riding simulator that consists of a Pentium 4 PC with a Windows XP operating system (see Figure 1 for an image of the HRT and examples of risky scenes).

The PC is placed on a base connected to an LCD monitor (1,024 × 768 resolution) displaying the virtual environment and to a chassis equipped with moped-like controls. For our procedure, a second screen was placed on a table behind participants who were seated on a moped-like seat approximately 80 cm from the HRT monitor. Two speakers, in addition to reproducing the acoustic effects of the engine and traffic noise, provided instructions to the active participants in the experimental group about the path they had to follow. Participants rode along the virtual courses using the moped-like controls, with a transmission set to automatic.

The same five courses, which took place on secondary roads, were employed in the first two sessions, while six courses, which took place on main roads, were administered in the last session. Each course included seven or eight risky scenes (depending on the course), for a total of 39 scenes in each of the first two sessions and 48 in the last one. The scenes represented hazardous road situations based on a European report classifying the most common motorcycle accident scenarios (Motorcycle Accidents In Depth Study [Maids] (2004)).

#### Skin Conductance Recording

Skin conductance was recorded with two Ag/AgCl electrodes filled with a K-Y lubricating jelly placed on the left foot, over the *abductor hallucis* muscle, in a position adjacent to the sole of the foot and midway between the base of the *hallucis* and a point beneath the ankle. This electrode placement conforms to the international guidelines (Boucsein et al., 2012) indicated in situations where participants need to use their hands for the task itself.

A Grass Model SCA1 skin conductance coupler provided a 0.5-V constant voltage across electrodes. The signal was amplified and filtered with a 10-Hz low-pass filter using a Grass CP122 AC/DC Strain Gage amplifier (Grass Instrument Co., W. Warwick, RI, United States). The amplifier—placed adjacent to the second screen, reproducing the output of the HRT monitor—returned a display of the ongoing values of the electrodermal activity. A video camera was employed to simultaneously record the electrodermal activity (the values on the amplifier display) and the riding performance (second monitor).

#### Procedure

For both groups, the entire procedure lasted for three sessions scheduled 1 week apart. Each session lasted approximately 45–60 min. At the beginning of the first session, after signing informed consent forms, each participant completed a questionnaire involving data related to their age and their driving and riding experience. Then, electrodes for skin conductance recording were attached.

With regard to the experimental group, the participants were instructed on how to use the HRT controls. Their task consisted of riding along the virtual courses, following the audibly vocalized advice, respecting the traffic rules, and avoiding crashes. The instructions also explained the importance of trying to avoid moving their feet during the task. A practice course (3 min in length, with no other road users in the virtual environment) was administered to enable participants to familiarize themselves with both the virtual environment and the HRT controls. All participants in the experimental group attempted the same five courses in the first two sessions, confronting a total of 39 potentially hazardous scenes in each session. In the last session, six new courses were administered, with a total of 48 scenes. Overall, each participant faced 126 hazardous scenes. As in the previous studies (Tagliabue and Sarlo, 2015; Tagliabue et al., 2017), the sequence of the courses was fixed for each session, proceeding from the easiest to the most difficult. The degree of difficulty was derived from the studies conducted by Miceli et al. (2008) and Settanni (2008).

The control group was asked to watch a simulation of some courses (five in the first two sessions and six in the third one) undertaken by an anonymous HRT rider and identify hazards. Whenever a passive participant detected a hazard, he or she pressed a button on the handlebar of the simulator. This detection task had the purpose of ensuring constant attention was paid to the video to avert the possibility of distraction. Given the purpose of the task, there was no need to record the responses. Each participant in the control group, matched to a same-gender participant in the experimental group, watched the replay of the performance of his or her paired participant in the corresponding sessions. At the end of each course, a 3-min rest was assigned to both the experimental and the control group to allow skin conductance to return to the baseline level.

#### Data Reduction and Coding

The coding procedure was based on the videos recorded by the camera (in which the electrodermal values displayed by the amplifier and the performance on the HRT were synchronized) and on the .csv files, provided by the simulator, that collected all the variables linked to the riding performance, with a sampling rate of 30 Hz. The electrodermal activity values were coded at this same sampling rate via analysis of the videos obtained for each participant in each session. In this way, each point of the physiological signal was matched with the behavioral variables provided in the .csv files. Among these variables, the HRT also provided an evaluation of participants’ riding safety for each scene. Possible scores ranged from A to D, depending on the distance between rider and collision, with A signifying that the participant’s behavior was safe enough to prevent any collisions, B indicating a slight increase in the risk of crash, C corresponding to a near-miss, and D representing to an actual crash. Thus, each performed scene received a score that represented its particular level of risk, depending on the rider’s behavior.

As in previous works (Tagliabue and Sarlo, 2015; Tagliabue et al., 2017), for each of the hazardous scenes, we identified a clue (i.e., a point on the path, in terms of *x*/*y* coordinates, after which hazard development began). As a result, we focused on two temporal windows: a baseline pre-clue window of 5 s and a 10-s post-clue window in which the hazard developed. Note here each participant was provided with the same clues, and this obtained for both the experimental and the control group.

As the participants in the experimental group could influence the development of the hazardous scenes via their riding behavior, the detection of the SCRs’ onset was not feasible in terms of timing. For instance, with a lower riding speed, approaching the hazard required more time. Therefore, the SCRs’ onset might appear to happen later because of a difference in the time taken to approach the hazard. As such, on the basis of the methodology employed in previous works (Tagliabue and Sarlo, 2015; Tagliabue et al., 2017), the mean level of the electrodermal activity in the baseline window was computed. Then, an SCR was detected as the first increase in amplitude of at least 0.05 μmho (Boucsein et al., 2012; Kinnear et al., 2013; Tagliabue and Sarlo, 2015; Tagliabue et al., 2017) in the post-clue window, with respect to the baseline. The time of SCR onset was then converted into its corresponding position on the path, in terms of the absolute value of *x* or *y* coordinates, depending on the dimension along which the participant was moving. Note that each individual change in the coordinates’ value corresponds to a change of approximately 1 m in the virtual environment. The onset was therefore computed in terms of spatial distance from the hazard: an anticipation in the SCR onset corresponded to a greater distance from the hazard. The coding procedure was the same for both groups in every session. Indeed, each participant in the control group watched the performance of his or her matched experimental participant, thereby being exposed to the same scenes, with the same *x*/*y* coordinates.

#### Design

To investigate the effectiveness of the three-session training, we conducted an ANOVA on the dependent variable accident’s percentage of participants who actively rode along the virtual courses (active group) with *Session* as a within-participants factor (three levels).

Moreover, a deeper analysis of the participants’ performance was conducted via a MANOVA on the percentage of scores (A, B, C, or D) attributed to each performed scene on the basis of the degree of risk generated by the participants’ behavior. Again, *Session* was the within-participants three-level factor.

To investigate whether psychophysiological responses paralleled improvements in riding performance, and to compare psychophysiological reactivity of the active group to that of the passive group, two ANOVAs were carried out on the dependent variable percentage of SCRs. The first was conducted on the overall SCR percentages (independently from the degree of risk of the scenes) and aimed at comparing the present results with the previous one attained by Tagliabue et al. (2017), with *Group* (active vs. passive) as a between-participants factor and *Session* (three levels) as a within-participants factor.

In addition, a further ANOVA with *Group* as a two-level between-participants factor and *Session* (three levels) and *Risk degree* (four levels: A, B, C, and D) as the two within-participants was run to provide more information via the fine-grained analysis of the pattern of psychophysiological changes with reference to the risk degree.

Skin conductance response percentages were calculated for each kind of scene, depending on the degree of risk, as the proportion of SCRs detected over the total scene in each risk degree category. In courses in which one kind of scene (A, B, C, or D) did not occur, depending on the participant performance in the active group, the missing data were replaced by the mean for the same risk category. This happened for one pair of participants for A scenes and five pairs of participants for D scenes.

Last, to test the impact of the three-session training on the anticipatory ability, we analyzed the onset of dependent variable SCR via an ANOVA with *Group* as a between-participants factor (active vs. passive) and *Session* (three levels) and *Risk degree* (four levels: A, B, C, and D) as within-participants factors.

*Post hoc* analyses using Bonferroni’s correction were conducted with α set at 0.05.

### Results

In the ANOVA on the percentage of accidents of the active group, the factor *Session* attained significance with *F*(2,34) = 107.46, *p* < 0.001 ($\eta_{p}^{2}$ = 0.86). Participants had 28% of accidents in the first session, 16% in the second session, and 4% in the third session. *Post hoc* tests showed that all comparisons were significant, thereby confirming that performance improved, both in the second session (which administered the same courses as the first session), and in the third session, when participants had to confront new courses.

**FIGURE 2:**
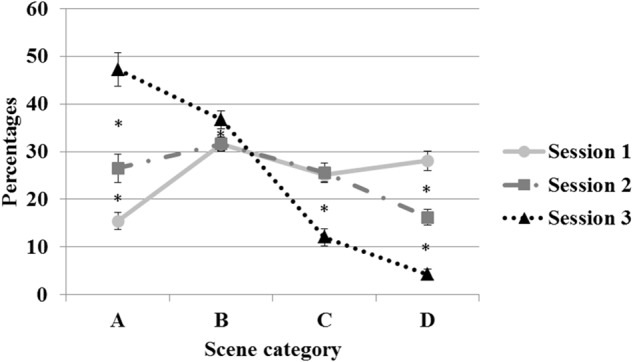
Percentages of the scores obtained during simulated moped riding in the three sessions. Error bars represent standard errors. Asterisks indicate the significant differences as attested by the *post hoc* tests.

In the MANOVA on the percentage of scene’s scores, the factor *Session* attained significance at the multivariate level, Wilks’ λ = 0.027, *F*(6,12) = 71.64, *p* < 0.001 ($\eta_{p}^{2}$ = 0.97). At the univariate level, the factor *Session* was significant for each score, with *F*(2,34) = 99.85, *p* < 0.001 ($\eta_{p}^{2}$ = 0.86) for the A score, *F*(2,34) = 5.01, *p* < 0.05 ($\eta_{p}^{2}$ = 0.23) for the B score, *F*(2,34) = 37.26, *p* < 0.001 ($\eta_{p}^{2}$ = 0.69) for the C score, and *F*(2,34) = 103.08, *p* < 0.001 ($\eta_{p}^{2}$ = 0.86) for the D score. The *post hoc* tests revealed that the percentage of A scores (i.e., totally safe scenes/performance) significantly increased between the first two sessions (15 vs. 26%) and from the second to the third session (47%). The percentage of D scores (i.e., accidents) decreased (28, 16, and 4% for the three sessions, respectively). With regard to B and C scores, no differences were found in the first two sessions, but in the third session, the percentage of both scores significantly differed (see Figure 2).

These results testify to the acquisition of learning as the training progressed. In the first session, participants drove in a way that generated a given degree of risk, as evidenced by the lower percentage of A scores relative to B and, even more clearly, to C and D scores that, it is worth noting, were attributable to scenes characterized by the development of a large amount of risk, or even the occurrence of an accident. The improvement in performance in the second session, which required participants to confront the same scenes, is demonstrated by a decrease in crashes. It can reasonably be considered that a certain number of scenes that had previously received a D score received a C or B score for the second session: the gradual modification of performance toward a notably safer level might be the reason why B and C scores appear to remain stable (as D scores become C scores, and C scores become B scores). Finally, this gradual improvement resulted in an unambiguous increase in the proportion of safe performances, as indicated by the enhanced percentage of A (safe performance) and B (low-risk performance) scores for the third session, in which participants faced six new (never before encountered) scenes. The effect of learning was even more apparent, given the significant concomitant reduction of C and D scores.

In the first of the ANOVAs on the SCR percentages, the factor *Group* and the factor *Session* attained significance with *F*(1,34) = 17.60, *p* < 0.001, ($\eta_{p}^{2}$ = 0.34), and *F*(2,68) = 7.47, *p* < 0.01, ($\eta_{p}^{2}$ = 0.18), respectively. Participants from the active group exhibited a higher percentage of SCRs, relative to the passive group (52 vs. 27%), and the SCR percentages decreased from 47%, for the first session to 39% for the second session and, finally, to 34% for the third session. The *post hoc* tests indicated that the SCR percentage for the first session was significantly higher than the subsequent percentages for the last two sessions.

In the second ANOVA on the SCR percentages, the factors *Group* and *Risk degree* attained significance with *F*(1,34) = 20.01, *p* < 0.001 ($\eta_{p}^{2}$ = 0.37) and *F*(3,102) = 53.22, *p* < 0.001 ($\eta_{p}^{2}$ = 0.61), respectively. In addition to the significant difference between the two groups, SCR percentages increased as the scenes became increasingly risky (32% in A scenes, 35% in B scenes, 46% in C scenes, and 60% in D scenes). The *post hoc* tests revealed that the SCR percentages in A and B scenes (safe or low-risk level) did not differ, but both were significantly different from SCR percentages in C and D scenes, which were characterized by a greater degree of risk development or the occurrence of an accident. The SCR percentages in C and D scenes were also significantly different from one another.

**FIGURE 3:**
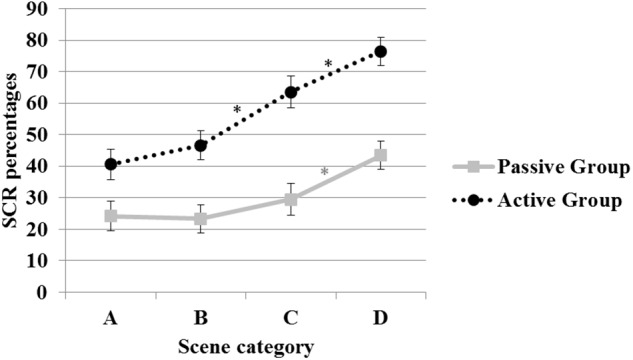
Percentages of SCRs shown by participants in each scene category, depending on the degree of risk developed. Error bars represent standard errors. Asterisks indicate the significant differences, as attested by the *post hoc* tests, within each group. The differences between groups are significant in each scene category.

Moreover, the *Group X Risk degree* and the *Session X Risk degree* interactions attained significance with *F*(3,102) = 6.01, *p* < 0.01 ($\eta_{p}^{2}$ = 0.15) and *F*(6,204) = 2.85, *p* < 0.05 ($\eta_{p}^{2}$ = 0.08), respectively. Concerning the *Group X Risk degree* interaction, as is evident in Figure 3, the *post hoc* test confirmed the significant difference between groups in each scene category. Moreover, in the active group, the SCR percentages increased in both C and D scenes, indicating that the psychophysiological reactivity paralleled the analogous rise in the degree of risk. Dissimilarly, in the passive group, a significant increase in SCR percentage was present only in cases of accident occurrence (relative to all other risk degrees).

**FIGURE 4:**
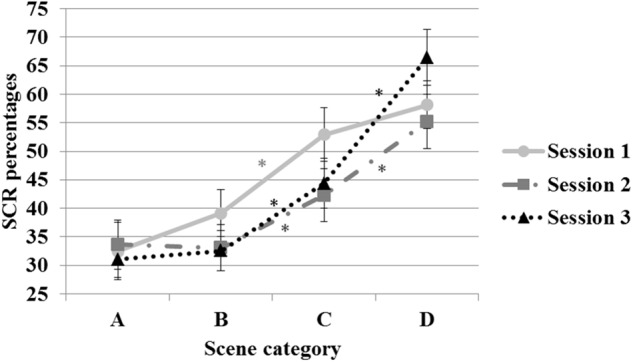
Percentages of SCRs shown by participants in each scene category, depending on the degree of risk developed. Error bars represent standard errors. Asterisks indicate the significant differences as attested by the *post hoc* tests.

As far as the *Session X Risk degree* interaction is concerned (see Figure 4), in the first session, A and B scenes elicited similar SCR percentages but were significantly different from C and D scenes, whereas SCR percentages of C and D scenes did not differ. In the second and third session, SCR percentages were higher in C than in B scenes and higher in D than in C scenes, as attested by the *post hoc* tests. Overall, this interaction suggests a better ability to discriminate the different degree of risk as the training progresses.

In the last ANOVA on the dependent variable SCR onset, the only main source of variance that reached significance was the factor *Session*, with *F*(2,68) = 11.94, *p* < 0.001 ($\eta_{p}^{2}$ = 0.26). None of the interactions reached significance. The *post hoc* tests showed that the onset in the second session (10 m) was different from the SCR onset in both the first (14 m) and third (16 m) sessions. The SCR onset of the first and third sessions (new scenes, never seen before) did not differ significantly. In other words, the anticipatory ability developed in the second session, when the same scenes had to be faced (as previously demonstrated in Tagliabue et al., 2017), disappeared in the third session when new, potentially risky scenes were performed or viewed by participants.

### Discussion

First, the results of the present paper confirm previous results that show performance improvement as training progresses. The fact that accident percentage decreases in the second session might be due to contextual learning, as the same course was administered in the first two sessions. However, the generalization of the learning acquired, at least at the behavioral level, has been demonstrated by the additional improvement recorded in the third session, in which six new courses were confronted. This improvement is substantiated not only by the reduction in accidents, but also by the overall improvement in performance. Participants who actively rode the moped simulator demonstrated safer behavior in the third session, as scenes in which they rode dangerously and incurred an accident decreased in the third session.

From a psychophysiological perspective, in accordance with previous reasoning, SCRs to potentially approaching hazards should indicate an implicit mechanism responsible for risk detection, in line with the dual processing system articulated by Slovic et al. (2007). Thus, one might wonder why SCR percentages should decrease, given that as the training progresses participants should have learned to react to hazards in a more effective way. Note that a reduction in SCR percentages between the first and second sessions was already observed in Tagliabue et al. (2017). The authors explained this result considering that, as the training develops, participants learn to drive safer, yielding a reduction in the number of near misses and accidents. In this case, there would be less need for the implicit system to react; thus, SCR percentages should decrease. In the present work, a third session was added to further test the hypothesis that the improvement derived from a safer riding style leads to a reduction of the number of hazards to detect and, consequently, of the amount of psychophysiological responses. The present data favor this explanation in that, in the third session (in which C and D scenes—i.e., near misses and accidents—decreased), the SCR percentage was lower than in the first session.

Moreover, these data replicate the results indicating greater emotional involvement in the active group, which showed higher SCR percentages than the passive group, thereby confirming the results of a study conducted by Tagliabue and Sarlo (2015) using a totally new sample.

The fine-grained analysis of the changes in SCR percentages based on the degree of risk involved in the scenes indicate that differences between the two groups are further evident in the modulation of psychophysiological reactivity pursuant to the risk degree (see Figure 3): unlike the active group, the enhancement of the SCR percentages in the passive group obtained only if an accident occurred may indicate a failure by the passive group to discriminate among different levels of hazard. This is a particularly compelling result as, while driving, the correct “categorization” of the risk degree might facilitate the selection of the most appropriate response.

Moreover, the results illustrated in Figure 4 indicate that accurate discrimination between no- or low-risk scenes, demonstrated by the psychophysiological reactivity, emerged in the second session and was maintained through the third session via transferring acquired learning to the new scenes, thereby providing partial evidence of generalization, at least in the simulated environment.

By contrast, with regard to anticipatory ability, our data did not confirm the generalization of this ability to scenes not yet encountered. Albeit negative, this result, if confirmed, provides important information about the requirements for road safety training, as it highlights the importance of extensive training aimed at exposing novice drivers and riders to as many different hazards as possible under the safer conditions of training (simulator or video clip viewing) to increase the probability that they will recognize such hazards in advance once they are actually on the road.

To summarize, active group participants showed performance improvement, and their learning seemed to generalize to new scenes, as they behaved more safely in both the second and third sessions. Moreover, they learned to discriminate among different degrees of risk via their implicit system (Slovic et al., 2007) and to generalize this achievement to the third session. By contrast, the anticipatory ability, in terms of SCR onset, did not appear to generalize to scenes not previously encountered. Results related to the passive group confirm previous results demonstrating that passive training involves the implicit system of hazard detection and recognition to a lesser extent.

## Conclusion

The main findings of the present research consist of demonstrating the following: (a) the experience of adverse consequence in simulated road scenarios yields an improvement in the ability to recognize the risk earlier when it is faced anew; (b) the psychophysiological correlates of this ability indicate that simulation is more effective than passive viewing of risky video clips; and (c) this anticipatory skill develops pursuant to prior experience, as predicted by the dual model of decision making (Slovic et al., 2007) and the somatic marker hypothesis of Damasio (1994).

The present results indicate a need to persist in attempts to develop training modules that permit exposure to the largest possible sample of road hazards before future motorcyclists actually take to the road to render them more capable of safely confronting each potential road risk. In this light, every effort to collect statistics, to map the greatest possible variations of the dynamics associated with the most common circumstances in which crashes occur and the attempt to replicate such dynamics via the simulators, must be firmly supported.

As noted, the hazard scenarios employed in the present research are drawn from the Motorcycle Accidents In Depth Study [Maids] (2004) accident statistics that include 921 situations representing a large amount of recorded motorcycle crashes across Europe. One potentially promising extension of this line of research could be the creation, for each of these prototypical conditions, of a certain number of variants (similar scenes with small differences), to induce learners and novice drivers to generalize, as much as possible, their acquired learning to new and different, but similar, scenes. To wit, the development of learning programs that enhance the probability of transferring the same anticipatory reactions to scenes not yet experienced by promoting generalization to several broader clusters of hazards could represent a further step toward the prevention of road accidents.

Given that experiencing an accident firsthand (albeit virtually) is more emotionally involving than simply viewing someone else’s accident, the greater impact of simulation relative to video clip viewing is in line with the evidence provided by studies in the field of affect heuristics that indicate that, the more vivid and affect-laden the scenarios, the more effective they are in influencing risk perception (Slovic et al., 2007).

The principal limitation of the present research is related to the generalizability of the observed effects to real on-road conditions. The use of simulators for driving/riding assessment and training is spreading in several countries. However, the benefits and disadvantages of this strategy remain controversial. Rosenbloom and Eldror (2014), for instance, did not find an overall improvement in on-road performance of newly licensed drivers trained with a driving simulator compared to novice drivers who received only the standard driving lessons. In fact, the former group showed a worsening in safe driving intention, probably due to overconfidence, and a slight reduction of headway events not associated with a reduction of their severity. The same group also showed an increase in the amount of brake pressure, which is interpreted by the authors as reflecting a less safe driving style. Goode et al. (2013) argued in favor of a certain amount of effectiveness associated with this technology when it is aimed at refining higher order cognitive skills, such as visual scanning and hazard perception; de Winter et al. (2012) cited the advantages and disadvantages of simulators and stressed the need for deeper investigation. More recently, evidence of correspondence between simulator and on-road driving parameters was reported by Branzi et al. (2017) by comparing driving speeds in on-road and simulated driving.

Despite the fact that the advantages and disadvantages of employing simulators are matters of ongoing debate (de Winter et al., 2012; Rosenbloom and Eldror, 2014), any reasonable enterprise aimed at improving the abilities of road users to prevent the development of risky situations should not be overlooked, especially as the available data seem to show that, over the last 5-year period, the goal of the EU, in terms of reducing the number of road deaths, appears far from being achieved (Adminaite et al., 2018). The present data suggest that, even though generalization from previously experienced road scenes is evident in the explicit behavior observed during the training of road users with limited experience, implicit learning requires prior exposure to each specific scenario. As such, any attempt to monitor and map conditions in which accidents occur and expose novice road users to the largest possible sample of such risky scenarios, so as to improve training programs, should be strongly encouraged.

## Ethics Statement

This study was carried out in accordance with the recommendations of guidelines for psychological research of the Associazione Italiana Psicologia (AIP) with written informed consent from all subjects. All subjects gave written informed consent in accordance with the Declaration of Helsinki.

The protocol was approved by the Ethical Committee for the Psychological Research of the University of Padua.

## Author Contributions

MT supervised data collection and contributed to statistical analyses and manuscript writing. EG conducted data collection, statistical analyses, and manuscript writing. MT, EG, and MS contributed to research planning, results’ discussion and revision of the paper.

## Conflict of Interest Statement

The authors declare that the research was conducted in the absence of any commercial or financial relationships that could be construed as a potential conflict of interest.
